# Isoelectric point region pI≈7.4 as a treasure island of abnormal proteoforms in blood

**DOI:** 10.15190/d.2016.14

**Published:** 2016-12-01

**Authors:** Mohammad Pirmoradian, Dag Aarsland, Roman A. Zubarev

**Affiliations:** Department of Medical Biochemistry and Biophysics, Karolinska Institutet, Stockholm, Sweden; Biomotif AB, Stockholm, Sweden; Alzheimer's Disease Research Centre, Department of Neurobiology, Care Sciences and Society, Karolinska Institutet, Stockholm, Sweden

**Keywords:** Isoelectric point, proteoforms, Alzheimer's disease, biomarkers

## Abstract

Theoretical distribution of isoelectric points (pI values) of human blood proteins exhibits multi-modality with a deep minimum in the range between pI 7.30 and 7.50. Considering that the pH of human blood is 7.4±0.1, normal forms of human proteins tend to eschew this specific pI region, thus avoiding charge neutrality that can result in enhanced precipitation. However, abnormal protein isoforms (proteoforms), which are the hallmarks and potential biomarkers of certain diseases, are likely to be found everywhere in the pI distribution, including this “forbidden” region. Therefore, we hypothesized that damaging proteoforms characteristic for neurodegenerative diseases are best detected around pI≈7.4. Blood serum samples from 14 Alzheimer's disease patients were isolated by capillary isoelectric focusing and analyzed by liquid chromatography hyphenated with tandem mass spectrometry. Consistent with the pI≈7.4 hypothesis, the 8 patients with fast memory decline had a significantly (p<0.003) higher concentration of proteoforms in the pI=7.4±0.1 region than the 6 patients with a slow memory decline. Moreover, protein compositions differed more from each other than for any other investigated pI region, providing absolute separation of the fast and slow decliner samples. The discovery of the “treasure island” of abnormal proteoforms in form of the pI≈7.4 region promises to boost biomarker development for a range of diseases.

## INTRODUCTION

Abnormal forms of human proteins (abnormal proteoforms)^1^ are often associated with human disease, such as Alzheimer's disease (AD), Amyotrophic lateral sclerosis, prion disease, Creutzfeldt–Jakob disease, Parkinson's disease (PD), amyloidosis, and a wide range of other disorders. Abnormal proteoforms arise due to misfolding (as in the above proteopathies), mutations and abnormal splicing^2^, excessive or unusual posttranslational modifications (PTMs), truncation^3^, cross-linking and aggregation, etc. Abnormal proteoforms are used as disease biomarkers^4^ and can serve as targets in therapies of the respective diseases.

Abnormalities in the protein primary or higher-order structure as well as in the PTM status result in changes in protein’s physico-chemical properties, such as molecular weight (MW) and isoelectric point pI. The latter is of particular analytical interest, as even a small change in amino acid sequence or 3D structure can result in a pI shift^5^. Protein pI can be estimated from the amino acid sequence using one of the computational approaches^6,7^. Several groups have computed proteome-wide pI distributions (calculated without the effects of 3D structure, mutations and PTMs), and discovered a general bimodality. The bimodal nature of proteome pI distribution results from the acidic Asp/Glu side chains and basic Lys/Arg side chains^3^. A common feature of many theoretical proteomes is the minimum (trough) between the acidic and basic peaks, found around pI≈7.4^8^. Some researchers believe that this trough is due to a combination of physico-chemical properties of amino acids, protein MW and length distribution^9^, while others find it to be consistent with the tendency of proteins to avoid pI equal to the media’s pH, as at such conditions the proteins acquire neutral overall charge and become prone to aggregation^10^. As experimentally-determined pH values for subcellular compartments differ significantly (lysosome 4.8; vacuole 5.3; Golgi 6.6; endoplasmic reticulum 7.1; cytoplasm 7.3; mitochondrion 7.5; nucleus 7.7; peroxisome 8.2)^11^, it was found that the average predicted pI for a subcellular compartment tends to deviate from the subcellular pH, consistent with mitigating against neutral-charge aggregation^10^.

**Figure 1:**
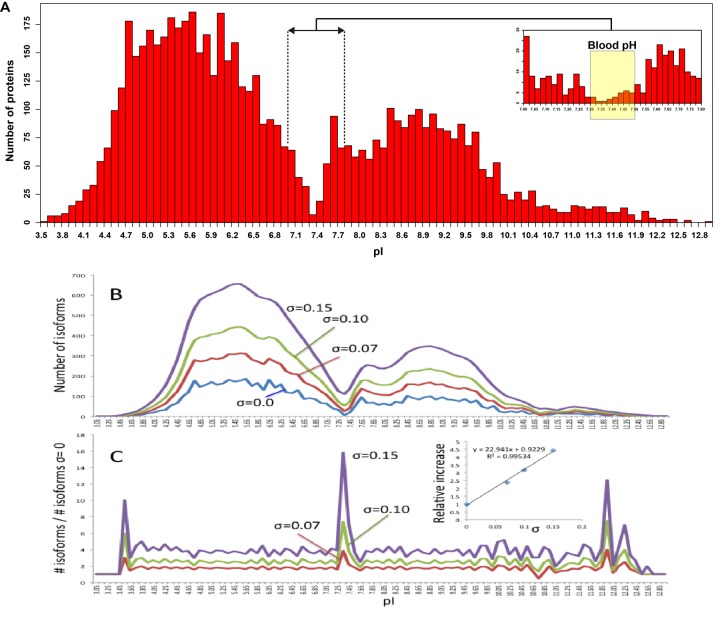
A. Theoretical pI distribution of 10,546 proteins in Plasma Proteome Database. B. The same as in A, assuming that every protein appears in a number of proteoforms with pIs distributed normally around the main proteoform with a standard deviation σ. C. The ratio between the plots in B and the plot at σ=0. Inset: increase in the number of proteoforms in the region of pI=7.4±0.05 compared to other pI regions as a function of σ.

Blood plasma is a major fluid compartment in human body, with a narrow normal range of pH (7.40±0.05)^12^. Thus the average pH of human blood is in the middle of the trough observed for the theoretical pI distribution of blood proteome (**Figure 1**[A]). Whether such a coincidence is by chance or by design, the density of normal proteoforms in the pI region 7.4±0.1 is very low (ca. ≈20 times lower than in the region around pI 5.3±0.1). Among few proteins with theoretical pI values close to 7.4 are TNF-receptor isoform TNFRSF1B and alpha-1 antiproteinase isoform SERPINA4. It should be noted, however, that the theoretical calculations can easily give an error of 0.2 pI units or larger^13^.

Abnormal proteoforms usually exhibit a shift in pI due to their deviating 3D structure^14^, mutations, PTMs, truncation as well as aggregation. Random shifting of the protein length while preserving the average amino acid composition characteristic for the proteome leads to fusion of the acidic and basic peaks of the pI distribution into one hump, with the trough at pI≈7.4 eliminated^10^. Increasing the protein length by, e.g., aggregation or complex formation with other proteins, also eliminates the trough^15^. Assuming that every gene product can appear in normal and a multitude of abnormal proteoforms, with the abnormals’ pIs being distributed according to a bell-shape curve around the pI of the normal form with a standard deviation σ, obtain model distributions of all proteoforms (**Figure 1**[B]) for which the ratio *R_a/n_* between the abnormal and normal proteoforms increases several-folds in the region pI≈7.4 compared to other regions (**Figure 1**[C], inset). Note that *R_a/n_* increases also for extreme pI values, but it is uncertain how extreme pI values of proteoforms can become in practice. The above observations led us to formulate the “pI-pH hypothesis” suggesting that the pI region near the pH of the local media is enriched with abnormal proteoforms. As blood is considered to be optimal bodily fluid for finding biomarkers due to its availability and low invasiveness of sampling, the pI-pH hypothesis, if correct, would open ways for more targeted biomarker discovery.

Testing the pI-pH hypothesis requires isolation of the proteins in a narrow pI range, which can be conveniently performed using isoelectric focusing (IEF). IEF in gel or capillary electrophoresis is often used as one of the dimensions of molecular separation (e.g., in 2D gel electrophoresis). IEF is also a broadly used fractionation technique in blood proteomics studies^16^. However, gel-based methods are tedious and not very well suited for subsequent detection and quantification of proteins with mass spectrometry (MS), due to inevitable losses associated with polypeptide extraction from the gel. Capillary-based methods either share the same drawbacks with gel-based methods or suffer from low sample capacity (<1 µg). Recently we have introduced a multijunction capillary isoelectric focusing (MJ-CIEF) device that combines high sample capacity (up to 100 µg), ease and speed of operation (<1 h) with low sample losses (<10%)^17,18^. This device helped to increase the depth of the proteome analysis of tryptic peptide mixtures^17^ and blood plasma proteins^18^. Here we employed the MJ-CIEF fractionator for testing the pI-pH hypothesis in serum of patients with neurodegenerative disorders.

**Figure 2:**
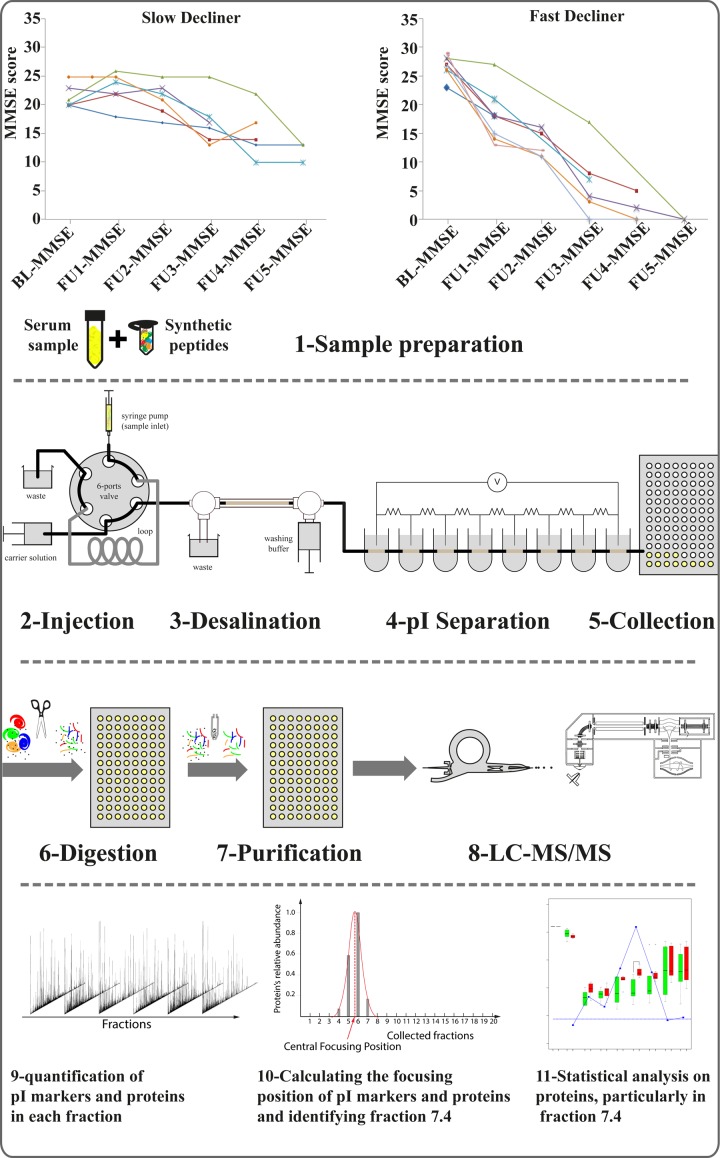
**Samples: **Mental decline in slow decliners (<2 MMSE units per year) and fast decliners (>5 MMSE units per year).** General workflow:** Each serum sample is spiked with a mixture of pI markers and is injected into the loop. Sample is washed in desalinator for 10 minutes and transferred to pI separation column. Proteins are fractionated by pI and 20 fractions are collected into a 96 well plate. Each fraction is digested and cleaned up prior to LC-MS/MS analysis. Upon peptide identification and quantification, the pI markers peptides are found and the fraction corresponding to pI≈7.4 is identified. The protein abundances in that fraction are statistically analyzed. The experimental pI value of each protein is determined from the abundances of that protein in each fraction.

In short, the workflow for hypothesis testing looks as follows (**Figure 2**). About 3 μL of serum spiked with a mixture of pI markers (synthetic peptides) are first injected into an online desalinator for removing salts interfering with pI separation. After 10 min of dialysis, the sample is transferred into the MJ-CIEF fractionator. The pI separation and focusing step lasts 30-60 min; upon its completion, 10-20 fractions are eluted and collected for further digestion, clean-up and LC-MS/MS analysis. In the acquired LC-MS/MS dataset, the marker peptides are identified and quantified, and thus the fraction representing the pI≈7.4 region is determined. Statistical analysis of the protein abundances will either support or contradict the hypothesis predictions, which are the following. First, the total protein abundance should be higher in the pI≈7.4 region in more severe disease than in healthier samples. Second, the relative protein abundances (normalized by the total protein content in each sample) should differ more between the disease states in that pI region than in other regions.

The above two predictions were tested on serum samples of patients diagnosed with Alzheimer’s disease (AD), the most common disorder causing senile dementia^19^. The onset of this neurodegenerative disease is accompanied by progressive decline in cognitive and functional abilities^20^. Considerable work has been devoted to finding AD biomarkers predicting the rate of disease progression^21^. The commonly used AD molecular biomarkers include increased tau protein and amyloid beta peptide (Aβ42) levels and tau hyperphosphorylation in cerebrospinal fluid (CSF), as well as amyloid beta lesions in brain, the latter detected by positron emission tomography^19^. A drawback of such examinations is the invasive lumbar puncture procedure to acquire CSF and the costly molecular imaging techniques that might not be available to all patients. Therefore, less invasive, more available and preferably more accurate molecular biomarkers are desired. While testing the pI-pH hypothesis, we will examine the possibility of using the pI≈7.4 region for discovering such biomarkers in form of abnormal proteoforms of common blood proteins. One of the reasons for proteoforms to appear in that forbidden region is aggregation, a process characteristic for AD as well as other neurodegenerative disorders^22^. The novel MJ-CIEF device is applied in the current study together with the optimized proteomics techniques developed previously in our lab^17,18,23^.

For testing the pI-pH hypothesis, patients from Western Norway diagnosed with probable AD and matched for gender (female) and age (77±6 years) were selected^24^. For each patient, the rate of mental decline was determined based on the Mini–mental state examination (MMSE) scores in the initial and follow-up examinations. According to the rate of MMSE score decline, the patients are usually classified as either slow (<2 MMSE units/year) or fast (≥2 MMSE units/year) decliners^25^. Here, the decline rates of slow decliners were <2 MMSE units/year rate, while those of fast decliners - ≥5 MMSE units/year.

## MATERIALS AND METHODS

*Blood Samples. *14 females from Western Norway diagnosed with probable AD were recruited (**Table 1**). Selection and diagnostic procedures were performed as previously described^24^. For each patient in the studied cohort, the MMSE score at the moment of blood sample collection was less than 26, and its regression was recorded at follow-up examinations 3 to 5 years after the first diagnosis. The difference between the MMSE scores was divided by the number of years between the examinations, to produce the average annual rate of MMSE decline. Six patients had the annual decline rate less than 2 MMSE units per year (slow decliners), while 8 patients had more than 5 MMSE units per year (fast decliners).

**Table 1:** Information on the patient samples used in the study

Cohort	Type	n	Gender	Age, year	MMSE baseline	MMSE decline, year-1
Slow decliner	ProbAD	6	female	78±9	22±2	(1.75±0.2)
Fast decliner	ProbAD	8	female	76±5	27±2	(6.3±1.3)

*pI markers. *Three peptides were synthesized (Peptide 2.0 Inc, USA) to cover the pH range around 7.4, and four more peptides – as controls with pIs in other regions (**Table 2**). Theoretical isoelectric point of each peptide was calculated by the ExPASSy online tool “Compute pI/MW”^26^. None of the peptides had a homologous sequence in human database, which is reflected in their low BLAST scores^27^. The hydrophobicity of peptides was calculated by the online tool “SSRCalc”^28^ to ensure elution from the LC column during the LC-MS/MS analysis. Peptides were resuspended in 0.1% formic acid (FA) to a final concentration of 20 mM. A mixture of all seven peptides was prepared and diluted to a final concentration of 1 µg/µL. 0.5 µL of the mixture was added to each sample containing 5 µL of serum.

**Table 2:** Synthetic peptides used as pI markers

Sequence	pI calc.	Rel. hydrophobicity calc.	BLAST max score
PYFSQAEYK	6.42	21.88	23.1
ELQLSHMIGK	6.85	24.10	22.3
HVIVHELHFHK	7.10	29.58	22.7
PFVGHVHDFHK	7.42	22.54	22.7
PHEWHIAHWHR	7.49	27.13	23.5
RPECQSWTCR	8.07	15.12	29.5
KPQFVSIGK	10.00	21.16	22.3

*Desalination and pI separation of blood proteins. *3 µL of each sample were loaded into the MJ-CIEF device. Desalting was performed by buffer exchange in the online desalinator^18^ using a washing solution composed of 0.5% Pharmalyte 6.6-7.6, 5% glycerol and 5mM dithiothreitol (DTT) in Milli-Q water at the flow rate of 500 µL/min for 10 min. Thereafter, the sample was transferred for pI separation into the capillary isoelectric focusing column. All external electrolytic buffer solutions (ammonium acetate and ammonium formate) were prepared at 10 mM concentration in degassed Milli-Q water. The buffers set the pH value in each vial, thus creating a nonlinear pH gradient across the whole device: from pH=6.5 in the anodic vial, followed by pH=6.7, 6.9, 7.1, 7.3, 7.5, 7.7 in the interval vials, and to pH=7.9 in the cathodic vial. Floating high voltage of up to -3 kV was applied in the regime of constant current of 40 µA. Isoelectric focusing was stopped when the voltage increase over time subsided, which usually took less than 50 min. Focused fractions were then mobilized and collected at the flow rate of 0.5 µL/min. A three-time stepwise releasing and refocusing was applied as described in detail previously^18^. For each sample, 20 fractions were collected.

*Protein digestion. *Proteins in each fraction were digested in solution as described before^29^. In brief, proteins were reduced with 10 mM DTT and alkylated with iodoacetamide at a final concentration of 10 mM. Proteins were digested with sequencing grade trypsin (Promega, USA) and incubated at 37 °C overnight. The digestion was terminated by the addition of 5% acetic acid (v/v), and the solution was rigorously vortexed for 5 min. All peptide mixtures were purified using acetonitrile elution from Hypersep™ Filter Plate C-18, (Thermo Scientific) and dried out in a SpeedVac to remove the solvent. The dry samples were resuspended in water with 0.1% formic acid and 2% acetonitrile.

*RPLC-MS/MS Analysis. *All fractions were analyzed in a random order. An EASY-Spray LC column (PepMap® RSLC, C18 material with 100 Å pores, 3 µm-bead-packed 15-cm column) from Thermo Scientific was used. The LC gradient lasted 54 min (total LC time – 70 min), while the flow rate was 250 nL/min. The gradient of buffer B (99.9% acetonitrile, 0.1% formic acid) was set as follows: 2% at the start, 5% at 6 min, 19% at 50 min and 30% at 59 min. The gradient was followed by a 5 min washing step at 95% buffer B. Mass spectra were acquired on an Orbitrap Q Exactive Plus mass spectrometer (Thermo Fisher Scientific) in a data-dependent manner using a top-10 MS/MS method. MS spectra were acquired at a resolution of 70,000 with a target value of 3E+06 ions or a maximum accumulation time of 250 ms in an m/z range from 300 to 2000. MS/MS spectra were acquired using HCD fragmentation with a normalized collision energy of 25 at a resolution of 17,500, with a target value of 2E+05 ions or a maximum accumulation time of 120 ms.

*Data analysis. *All 280 raw data files were processed by MaxQuant v. 1.5.0.25, which performed peptide and protein identification and quantification^30^. As a sequence database, the International Protein Index (human version 3.87; 91,464 protein sequences) was used. Mass tolerance for precursor ions in MS/MS search was 20 ppm in the initial search and 6 ppm in the main search. Cysteine carbamidomethylation was selected as a fixed modification, while acetylation of the protein N-terminus, oxidation of methionine and deamidation of asparagine and glutamine were selected as variable modifications. Up to two missed cleavages were allowed in the matched peptides. The results were filtered to a 1% false discovery rate at both protein and peptide levels^31^. Further analysis was performed of the data in the output file proteinGroups.txt. The MaxQuant-reported ‘LFQ-intensity’ of each protein was taken as relative protein abundance. Statistical tests and calculations were done using Microsoft Excel and R. The focusing position for each protein on the pI scale was determined as the weighted average of the iBAQ-intensity values in individual fractions, as previously described^17^.

The in-silico analysis of theoretical pI values in blood proteome was performed based on the Plasma Proteome Database^32^. The amino acid sequences of all 10,546 proteins were retrieved from the Ensembl database^33^. The proteins’ theoretical isoelectric points were calculated using the “seqinR” package in R^34^. The results were plotted using R in form of a pI histogram.

## RESULTS AND DISCUSSIONS

### Label free quantification of blood proteins

**Figure 3:**
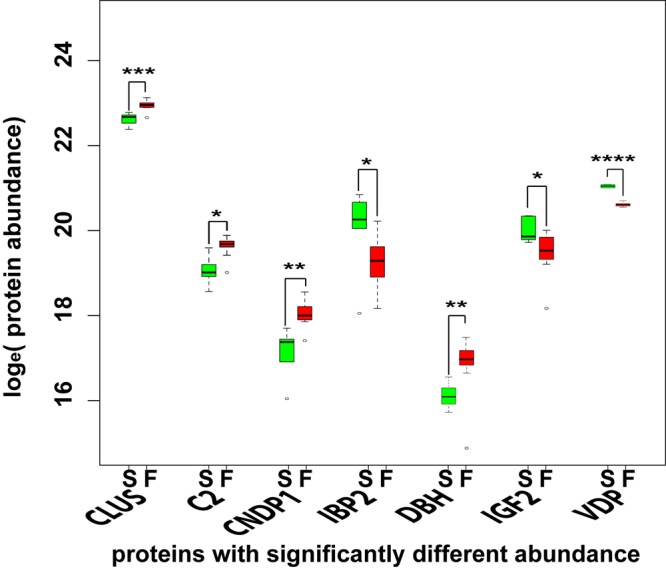
Protein abundance differences between fast (F) and slow (S) decliners Each box represents abundances of blood serum proteins: Clusterin (CLUS), Complement component 2 (C2), Beta-Ala-His dipeptidase (CNDP1), Insulin-like growth factor-binding protein 2 (IBP2), Dopamine beta-hydroxylase (DBH), Insulin-like growth factor 2 (IGF2), and Vitamin D-binding protein (VDP).

Analysis of all LC-MS/MS datasets resulted in quantification of 650 protein groups that passed 1% false discovery rate (FDR) threshold at both peptide and protein levels. To assess the utility of the fractionated proteome for biomarker discovery, the normalized protein abundances were compared between the two groups of patients. Top ten proteins with significant abundance changes (p<0.05) were selected for detailed consideration. Several of these proteins are known to be involved in neurodegenerative disease pathways, e.g., clusterin^35,36^, beta-Ala-His dipeptidase^37,38^, dopamine beta-hydroxylase (DBH), insulin-like growth factor and insulin-like growth factor-binding protein^39,40^, vitamin D-binding protein (VDP), as well as members of the complement system^41^ and serpin family^40,42^ (Figure 3).

There is extensive knowledge implicating the above proteins in AD-related processes. Dopamine beta-hydroxylase (its level is found elevated in fast decliners) is an enzyme that synthesizes norepinephrine from dopamine. Decrease in the activity of this enzyme has been found in cerebrospinal cortex of the AD patients^43^. Several genetic studies have reported that polymorphism in this gene as well as the proximal region of it promoters are associated with the disease^44^.

Vitamin D-binding protein is a multifunctional molecule whose level is elevated in CSF of AD patients^45^. Here, VDP level is significantly reduced in fast decliners compared to slow decliners (Figure 3). The role of VDP in binding to and active removing of actin filaments in AD has been suggested^46^. Other studies reported the role of VDP in suppression of Aβ-mediated pathologies^47^.

Transgenic mice that over-express the APP Swedish mutation exhibit elevated levels of insulin-like growth factor 2 (IGF2), insulin-like growth factor 2 binding protein (IGFBP2), and ectonucleotide pyrophosphatase (ENP2) well before the onset of Aβ deposition^48,49^. Both IGF2 and the IGFBP2 have been reported as markers in patients with neurodegenerative diseases^39^. A possible stimulatory effect of soluble Aβ peptide in activation of these proteins in AD brain have been suggested in an in vivo study^39^. The level of IGF2 is found to be significantly reduced in fast decliners compared to slow decliners (**Figure 3**).

Clusterin has been found in amyloid plaques^35^. Clusterin can also play a chaperone role in degradation of the Aβ peptide^35^. Clusterin has been suggested as a biomarker candidate for multiple sclerosis, with lower levels of protein in patients compared to healthy controls^50^. However, in AD and PD the clusterin abundance has been found elevated^51^. Here, the clusterin abundance is found to be significantly higher in fast decliners than in slow decliners.

Beta-Ala-His dipeptidase (found here at elevated levels in fast decliners) has been implicated in regulation of the immune response^37^.

### Narrow PI≈7,4 region

**Figure 4:**
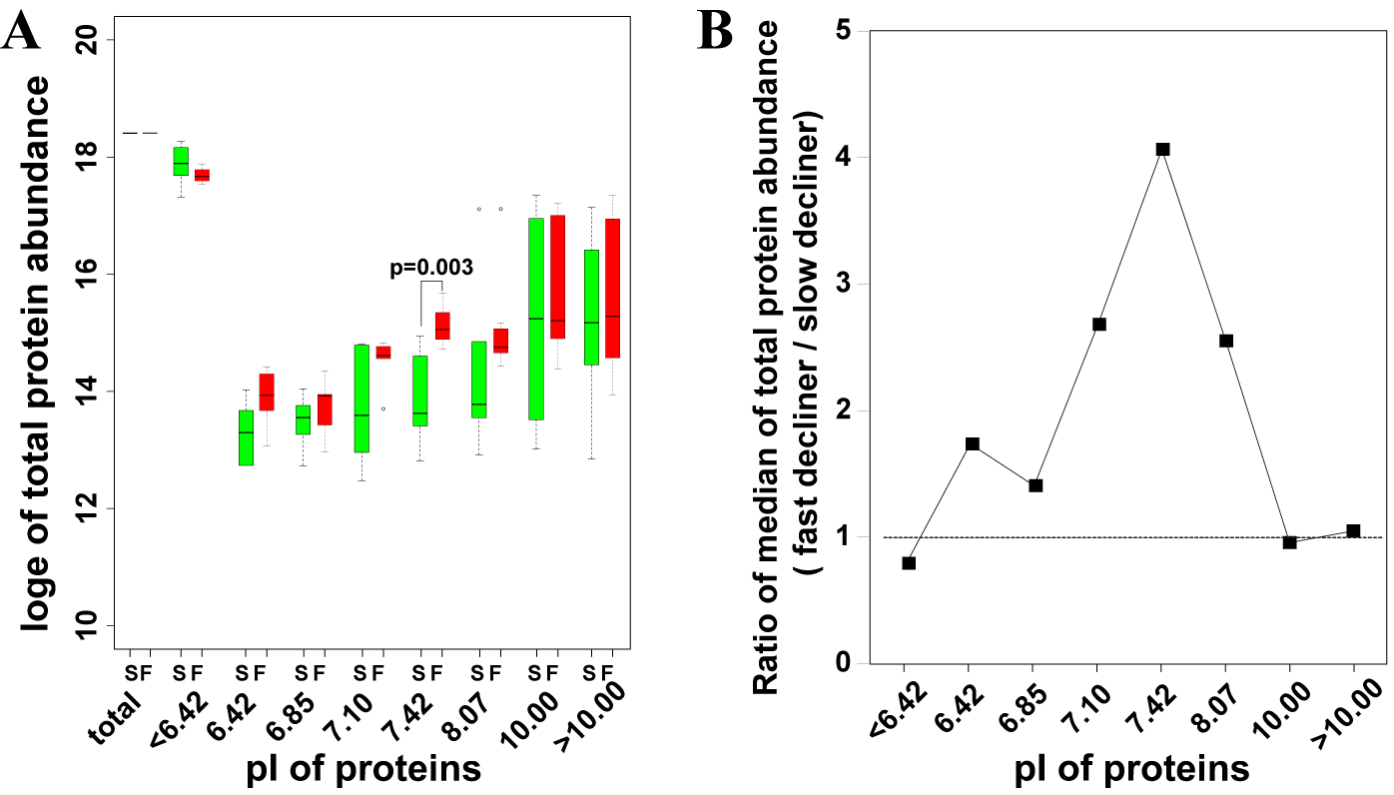
A. Total abundance of proteins in each pI-fraction: slow decliners (green) and fast decliners (red); B. The ratio between the medians of total proteins abundances in each fraction. The highest ratio is in the fraction pI≈7.4.

The fraction with 7.1 < pH < 7.5 was identified by means of spiked pI markers (**Figure 2**). The total abundance of the proteins in this fraction was compared between the two groups of patients. As a reference value, the same comparison was also performed for the neighboring fractions. A significantly changed (elevated) total protein concentration was only found in the pI≈7.4 fraction, in agreement with our hypothesis (**Figure 4**). The median total protein abundance in this region was more than 4 times higher in fast decliners than in slow decliners. The protein concentration of this narrow pH region could therefore be used for predicting the speed of mental decline in AD patients.

**Figure 5:**
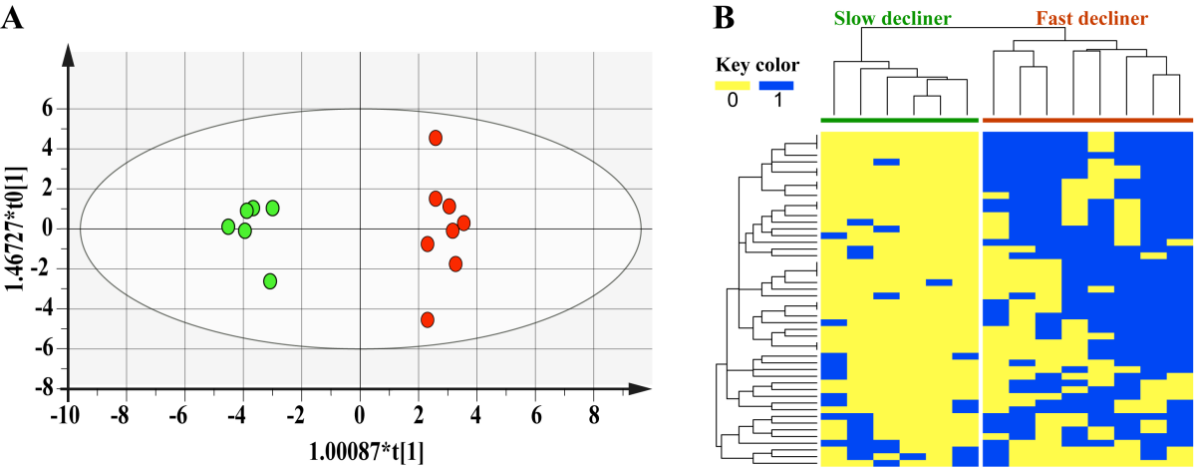
A. OPLS-DA model built based on proteins abundances in the region pI≈7.4. Green circles - slow decliners; red circles - fast decliners; B. Heatmap of unsupervised clustering of samples based on proteins abundances at p≈7.4. Each row represents a protein that was observed in at least one of the samples. Each column is a sample: green on top - slow decliners, and red on top - fast decliners. Blue - the central focusing position of that protein was at pI≈7.4; yellow - elsewhere.

An OPLS-DA model built based on protein abundances in the pI≈7.4 region resulted in perfect separation of the patient groups, as shown in **Figure 5**[A]. Most proteins contributing to the separation are either members of immunoglobulin family, or have known roles in AD^52,53^, including carbonic anhydrase II, glucokinase, transthyretin, insulin-like growth factor binding protein 3, ganglioside GM2 activator, apolipoprotein C-I, calmodulin-like protein 5, and caspase-14.

Carbonic anhydrase II has a role in regulating the pH and also synaptic transportation. Several studies reported the elevated levels of this protein in both Down syndrome and AD^8^. Glucokinase triggers the shifts in metabolism or cell function in response to glucose level. The correlation between the expression of this gene and the mechanisms of insulin action and insulin resistance has been reported in neurodegenerative diseases, including AD^54^. The role of transthyretin (TTR) in AD is reported to be the same as for vitamin D binding protein, which is detoxification of neuronal cells from actin fibrils and amyloid beta^48^. In transgenic mouse models, the TTR level increased before appearance of amyloid plaques, suggesting TTR to be an early biomarker of AD^48,49^. As for ganglioside GM2 activator, the level and density of GM2 increase in neurodegenerative diseases^54^. Calmodulin is one of the primary calcium signal transducer, which regulates the calcium balance in the cell. Many studies suggested the disruption of calcium signaling as the cause of neuronal apoptosis in AD, and reported the role of Calmodulin as well as Calmodulin binding domain in AD^55^.

In the generated OPLS-DA model, R^2^ (fitness) was 97%, and Q^2^ (predictive power) was 72%. The high predictive power demonstrates the analytical potential of the pI≈7.4 isolation approach.

For proteins discussed above, the theoretical pI based on the amino acid sequence is listed in **Table 3**. Most proteins here perform carrier function, such as transthyretin, apolipoprotein C-I and insulin-like growth factor binding protein 3 and calmodulin-like protein 5. Thus the pI-shift to ≈7.4 could be due to the coupled form of the protein with a carried compound. For proteins such as caspase and complement factors, concatenation can also be the cause of the pI shift.

**Table 3:** Theoretical and experimentally determined isoelectric points of proteins observed in the region pI≈7.4

Protein	Theoretical pI	Median pI in slow decliners	Median pI in fast decliners
Carbonic anhydrase 2	6.87	Not observed	7.4
Apolipoprotein C-I	8.01	< 6.4	7.4
Transthyretin	5.49	7.1	7.4
Caspase-14	6.09	< 6.4	7.4
Serpin B3	6.35	< 6.4	7.4
Glucokinase	5.1	7.1	7.4
IGFBP-3	9.03	7.1	7.4
Ganglioside GM2 activator	5.17	7.1	7.4
Calmodulin-like protein 5	5.34	7.1	7.4

We were unable to detect any PTMs on the identified peptides that could account for the observed pI shift of the proteins. Therefore, further analyses, e.g., at the level of intact proteins and protein complexes, are needed to resolve this issue.

## CONCLUSION

Here we tested the pI-pH hypothesis by fractionating blood serum proteins using capillary isoelectric focusing. pI-based fractionation prior to shotgun proteomics increased the depth of proteome analysis, as expected. Consistent with the pI-pH hypothesis, the protein concentration in the pI=7.4±0.1 region turned out to be significantly higher (p<0.003) in patients with fast memory decline than in slow decliners. The proteins identified and quantified in this pI region provided an OPLS-DA model with excellent predictive power. This protein panel encompasses several previously reported AD biomarkers as well as a few novel biomarker candidates. At this moment it is unclear why these proteins appear around pI≈7.4, since their theoretical pIs are well outside this region. Aggregation, truncation and posttranslational modifications are the prime candidates. Further investigation is needed for studying the correlation between the protein concentration at pI≈7.4 and the disease state, as well as the specificity of this marker for different neurodegenerative diseases. But it is now certain that the pI≈7.4 region represents a true “treasure island” of abnormal proteoforms.

## Bullet Points

**◊ The concept of a proteoform has recently gained much attention. Among the human proteoforms, the abnormal ones are of particular research interest because of their link to disease and diagnostics. Here we provide a general method of enriching the abnormal proteoforms compared to normal forms of the same protein as well as other proteins.**

**◊ The method works for human blood as well as tissues, and it is based on the common tendency of normal protein forms to avoid the isoelectric point region around pI≈7.4 (pH of blood and many other bodily liquids). Thus, isolation of this pI region automatically enriches abnormal proteoforms.**

**◊ Our method is both novel and immediately useful. It can be easily implemented using, e.g., the novel pI-Trap device (Biomotif AB, Sweden); however, other isoelectric focusing devices can also be employed.**

**◊ Here we demonstrate the utility of the pI≈7.4 method in prognostics of Alzheimer’s disease. However, the method could potentially be applied to any disease involving abnormal proteoforms, including other neurodegenerative diseases.**

## References

[1] Smith Lloyd M, Kelleher Neil L (2013). Proteoform: a single term describing protein complexity.. Nature methods.

[2] Tazi Jamal, Bakkour Nadia, Stamm Stefan (2009). Alternative splicing and disease. Biochimica et Biophysica Acta (BBA) - Molecular Basis of Disease.

[3] Weiller Georg F., Caraux Gilles, Sylvester Nicole (2004). The modal distribution of protein isoelectric points reflects amino acid properties rather than sequence evolution. PROTEOMICS.

[4] Théberge Roger, Infusini Giuseppe, Tong Weiwei, McComb Mark E, Costello Catherine E (2011). Top-Down Analysis of Small Plasma Proteins Using an LTQ-Orbitrap. Potential for Mass Spectrometry-Based Clinical Assays for Transthyretin and Hemoglobin.. International journal of mass spectrometry.

[5] Righetti Pier Giorgio, Sebastiano Roberto, Citterio Attilio (2013). Capillary electrophoresis and isoelectric focusing in peptide and protein analysis.. Proteomics.

[6] Halligan Brian D, Ruotti Victor, Jin Weihong, Laffoon Scott, Twigger Simon N, Dratz Edward A (2004). ProMoST (Protein Modification Screening Tool): a web-based tool for mapping protein modifications on two-dimensional gels.. Nucleic acids research.

[7] Sillero Antonio, Maldonado Andres (2006). Isoelectric point determination of proteins and other macromolecules: oscillating method.. Computers in biology and medicine.

[8] Jang Bong Geom, Yun Sang-Moon, Ahn Kyungsook, Song Ju Hee, Jo Sangmee A, Kim Young-Yul, Kim Doh Kwan, Park Moon Ho, Han Changsu, Koh Young Ho (2010). Plasma carbonic anhydrase II protein is elevated in Alzheimer's disease.. Journal of Alzheimer's disease : JAD.

[9] Wu Songfeng, Wan Ping, Li Jianqi, Li Dong, Zhu Yunping, He Fuchu (2006). Multi-modality of pI distribution in whole proteome.. Proteomics.

[10] Chan Pedro, Lovrić Josip, Warwicker Jim (2006). Subcellular pH and predicted pH-dependent features of proteins.. Proteomics.

[11] Chan Pedro, Warwicker Jim (2009). Evidence for the adaptation of protein pH-dependence to subcellular pH.. BMC biology.

[12] Waugh A (2007). Ross and Wilson: Anatomy and Physiology in Health and Illness, 10th edition.

[13] Halligan Brian D (2009). ProMoST: a tool for calculating the pI and molecular mass of phosphorylated and modified proteins on two-dimensional gels.. Methods in molecular biology (Clifton, N.J.).

[14] Chatterjee R, Welty E V, Walder R Y, Pruitt S L, Rogers P H, Arnone A, Walder J A (1986). Isolation and characterization of a new hemoglobin derivative cross-linked between the alpha chains (lysine 99 alpha 1----lysine 99 alpha 2).. The Journal of biological chemistry.

[15] Carugo Oliviero (2007). Isoelectric points of multi-domain proteins.. Bioinformation.

[16] Pernemalm Maria, Lehtiö Janne (2014). Mass spectrometry-based plasma proteomics: state of the art and future outlook.. Expert review of proteomics.

[17] Pirmoradian Mohammad, Zhang Bo, Chingin Konstantin, Astorga-Wells Juan, Zubarev Roman A (2014). Membrane-assisted isoelectric focusing device as a micropreparative fractionator for two-dimensional shotgun proteomics.. Analytical chemistry.

[18] Pirmoradian Mohammad, Astorga-Wells Juan, Zubarev Roman A (2015). Multijunction Capillary Isoelectric Focusing Device Combined with Online Membrane-Assisted Buffer Exchanger Enables Isoelectric Point Fractionation of Intact Human Plasma Proteins for Biomarker Discovery.. Analytical chemistry.

[19] Hye Abdul, Riddoch-Contreras Joanna, Baird Alison L, Ashton Nicholas J, Bazenet Chantal, Leung Rufina, Westman Eric, Simmons Andrew, Dobson Richard, Sattlecker Martina, Lupton Michelle, Lunnon Katie, Keohane Aoife, Ward Malcolm, Pike Ian, Zucht Hans Dieter, Pepin Danielle, Zheng Wei, Tunnicliffe Alan, Richardson Jill, Gauthier Serge, Soininen Hilkka, Kłoszewska Iwona, Mecocci Patrizia, Tsolaki Magda, Vellas Bruno, Lovestone Simon (2014). Plasma proteins predict conversion to dementia from prodromal disease.. Alzheimer's & dementia : the journal of the Alzheimer's Association.

[20] Humpel Christian (2011). Identifying and validating biomarkers for Alzheimer's disease. Trends in Biotechnology.

[21] Moya-Alvarado Guillermo, Gershoni-Emek Noga, Perlson Eran, Bronfman Francisca C (2016). Neurodegeneration and Alzheimer's disease (AD). What Can Proteomics Tell Us About the Alzheimer's Brain?. Molecular & cellular proteomics : MCP.

[22] Lista Simone, Faltraco Frank, Prvulovic David, Hampel Harald (2013). Blood and plasma-based proteomic biomarker research in Alzheimer's disease.. Progress in neurobiology.

[23] Chingin Konstantin, Astorga-Wells Juan, Pirmoradian Najafabadi Mohammad, Lavold Thorleif, Zubarev Roman A (2012). Separation of polypeptides by isoelectric point focusing in electrospray-friendly solution using a multiple-junction capillary fractionator.. Analytical chemistry.

[24] Aarsland Dag, Rongve Arvid, Nore Sabine Piepenstock, Skogseth Ragnhild, Skulstad Siri, Ehrt Uwe, Hoprekstad Dagne, Ballard Clive (2008). Frequency and case identification of dementia with Lewy bodies using the revised consensus criteria.. Dementia and geriatric cognitive disorders.

[25] Doody R S, Massman P, Dunn J K (2001). A method for estimating progression rates in Alzheimer disease.. Archives of neurology.

[26] (2005). The Proteomics Protocols Handbook.

[27] Altschul Stephen F. (1993). A protein alignment scoring system sensitive at all evolutionary distances. Journal of Molecular Evolution.

[28] Krokhin Oleg V., Spicer Vic (2010). Predicting Peptide Retention Times for Proteomics. Current Protocols in Bioinformatics.

[29] Pirmoradian Mohammad, Budamgunta Harshavardhan, Chingin Konstantin, Zhang Bo, Astorga-Wells Juan, Zubarev Roman A. (2013). Rapid and Deep Human Proteome Analysis by Single-dimension Shotgun Proteomics. Molecular & Cellular Proteomics.

[30] Cox Jürgen, Mann Matthias (2008). MaxQuant enables high peptide identification rates, individualized p.p.b.-range mass accuracies and proteome-wide protein quantification.. Nature biotechnology.

[31] Cox Jürgen, Hein Marco Y, Luber Christian A, Paron Igor, Nagaraj Nagarjuna, Mann Matthias (2014). Accurate proteome-wide label-free quantification by delayed normalization and maximal peptide ratio extraction, termed MaxLFQ.. Molecular & cellular proteomics : MCP.

[32] Nanjappa Vishalakshi, Thomas Joji Kurian, Marimuthu Arivusudar, Muthusamy Babylakshmi, Radhakrishnan Aneesha, Sharma Rakesh, Ahmad Khan Aafaque, Balakrishnan Lavanya, Sahasrabuddhe Nandini A, Kumar Satwant, Jhaveri Binit Nitinbhai, Sheth Kaushal Vinaykumar, Kumar Khatana Ramesh, Shaw Patrick G, Srikanth Srinivas Manda, Mathur Premendu P, Shankar Subramanian, Nagaraja Dindagur, Christopher Rita, Mathivanan Suresh, Raju Rajesh, Sirdeshmukh Ravi, Chatterjee Aditi, Simpson Richard J, Harsha H C, Pandey Akhilesh, Prasad T S Keshava (2014). Plasma Proteome Database as a resource for proteomics research: 2014 update.. Nucleic acids research.

[33] Cunningham Fiona, Amode M Ridwan, Barrell Daniel, Beal Kathryn, Billis Konstantinos, Brent Simon, Carvalho-Silva Denise, Clapham Peter, Coates Guy, Fitzgerald Stephen, Gil Laurent, Girón Carlos García, Gordon Leo, Hourlier Thibaut, Hunt Sarah E, Janacek Sophie H, Johnson Nathan, Juettemann Thomas, Kähäri Andreas K, Keenan Stephen, Martin Fergal J, Maurel Thomas, McLaren William, Murphy Daniel N, Nag Rishi, Overduin Bert, Parker Anne, Patricio Mateus, Perry Emily, Pignatelli Miguel, Riat Harpreet Singh, Sheppard Daniel, Taylor Kieron, Thormann Anja, Vullo Alessandro, Wilder Steven P, Zadissa Amonida, Aken Bronwen L, Birney Ewan, Harrow Jennifer, Kinsella Rhoda, Muffato Matthieu, Ruffier Magali, Searle Stephen M J, Spudich Giulietta, Trevanion Stephen J, Yates Andy, Zerbino Daniel R, Flicek Paul (2015). Ensembl 2015.. Nucleic acids research.

[34] Charif Delphine, Lobry Jean R. (2007). SeqinR 1.0-2: A Contributed Package to the R Project for Statistical Computing Devoted to Biological Sequences Retrieval and Analysis. Structural Approaches to Sequence Evolution.

[35] Schjeide Brit-Maren M, Schnack Cathrin, Lambert Jean-Charles, Lill Christina M, Kirchheiner Julia, Tumani Hayrettin, Otto Markus, Tanzi Rudolph E, Lehrach Hans, Amouyel Philippe, von Arnim Christine A F, Bertram Lars (2011). The role of clusterin, complement receptor 1, and phosphatidylinositol binding clathrin assembly protein in Alzheimer disease risk and cerebrospinal fluid biomarker levels.. Archives of general psychiatry.

[36] Thambisetty Madhav, Simmons Andrew, Velayudhan Latha, Hye Abdul, Campbell James, Zhang Yi, Wahlund Lars-Olof, Westman Eric, Kinsey Anna, Güntert Andreas, Proitsi Petroula, Powell John, Causevic Mirsada, Killick Richard, Lunnon Katie, Lynham Steven, Broadstock Martin, Choudhry Fahd, Howlett David R, Williams Robert J, Sharp Sally I, Mitchelmore Cathy, Tunnard Catherine, Leung Rufina, Foy Catherine, O'Brien Darragh, Breen Gerome, Furney Simon J, Ward Malcolm, Kloszewska Iwona, Mecocci Patrizia, Soininen Hilkka, Tsolaki Magda, Vellas Bruno, Hodges Angela, Murphy Declan G M, Parkins Sue, Richardson Jill C, Resnick Susan M, Ferrucci Luigi, Wong Dean F, Zhou Yun, Muehlboeck Sebastian, Evans Alan, Francis Paul T, Spenger Christian, Lovestone Simon (2010). Association of plasma clusterin concentration with severity, pathology, and progression in Alzheimer disease.. Archives of general psychiatry.

[37] Kroksveen A C, Opsahl J A, Aye T T, Ulvik R J, Berven F S (2011). Proteomics of human cerebrospinal fluid: discovery and verification of biomarker candidates in neurodegenerative diseases using quantitative proteomics.. Journal of proteomics.

[38] Zürbig Petra, Jahn Holger (2012). Use of proteomic methods in the analysis of human body fluids in Alzheimer research.. Electrophoresis.

[39] de la Monte Suzanne M (2012). Brain insulin resistance and deficiency as therapeutic targets in Alzheimer's disease.. Current Alzheimer research.

[40] Doecke James D, Laws Simon M, Faux Noel G, Wilson William, Burnham Samantha C, Lam Chiou-Peng, Mondal Alinda, Bedo Justin, Bush Ashley I, Brown Belinda, De Ruyck Karl, Ellis Kathryn A, Fowler Christopher, Gupta Veer B, Head Richard, Macaulay S Lance, Pertile Kelly, Rowe Christopher C, Rembach Alan, Rodrigues Mark, Rumble Rebecca, Szoeke Cassandra, Taddei Kevin, Taddei Tania, Trounson Brett, Ames David, Masters Colin L, Martins Ralph N (2012). Blood-based protein biomarkers for diagnosis of Alzheimer disease.. Archives of neurology.

[41] Bergamaschini L, Canziani S, Bottasso B, Cugno M, Braidotti P, Agostoni A (1999). Alzheimer's beta-amyloid peptides can activate the early components of complement classical pathway in a C1q-independent manner.. Clinical and experimental immunology.

[42] Liao Pao-Chi, Yu Lung, Kuo Chih-Chieh, Lin Chingju, Kuo Yu-Min (2007). Proteomics analysis of plasma for potential biomarkers in the diagnosis of Alzheimer's disease.. Proteomics. Clinical applications.

[43] Cross A J, Crow T J, Perry E K, Perry R H, Blessed G, Tomlinson B E (1981). Reduced dopamine-beta-hydroxylase activity in Alzheimer's disease.. British medical journal (Clinical research ed.).

[44] Mustapic Maja, Maihofer Adam X, Mahata Manjula, Chen Yuqing, Baker Dewleen G, O'Connor Daniel T, Nievergelt Caroline M (2014). The catecholamine biosynthetic enzyme dopamine β-hydroxylase (DBH): first genome-wide search positions trait-determining variants acting additively in the proximal promoter.. Human molecular genetics.

[45] Bishnoi Ram J, Palmer Raymond F, Royall Donald R (2015). Vitamin D binding protein as a serum biomarker of Alzheimer's disease.. Journal of Alzheimer's disease : JAD.

[46] Gressner Olav A, Schifflers Marie-Claire, Kim Philipp, Heuts Leo, Lahme Birgit, Gressner Axel M (2009). Questioning the role of actinfree Gc-Globulin as actin scavenger in neurodegenerative central nervous system disease: relationship to S-100B levels and blood-brain barrier function.. Clinica chimica acta; international journal of clinical chemistry.

[47] Moon M, Song H, Hong H J, Nam D W, Cha M-Y, Oh M S, Yu J, Ryu H, Mook-Jung I (2013). Vitamin D-binding protein interacts with Aβ and suppresses Aβ-mediated pathology.. Cell death and differentiation.

[48] Stein Thor D, Johnson Jeffrey A (2002). Lack of neurodegeneration in transgenic mice overexpressing mutant amyloid precursor protein is associated with increased levels of transthyretin and the activation of cell survival pathways.. The Journal of neuroscience : the official journal of the Society for Neuroscience.

[49] Lazarov Orly, Robinson John, Tang Ya-Ping, Hairston Ilana S, Korade-Mirnics Zeljka, Lee Virginia M-Y, Hersh Louis B, Sapolsky Robert M, Mirnics Karoly, Sisodia Sangram S (2005). Environmental enrichment reduces Abeta levels and amyloid deposition in transgenic mice.. Cell.

[50] Comabella Manuel, Fernández Marta, Martin Roland, Rivera-Vallvé Stephanie, Borrás Eva, Chiva Cristina, Julià Eva, Rovira Alex, Cantó Ester, Alvarez-Cermeño Jose Carlos, Villar Luisa María, Tintoré Mar, Montalban Xavier (2010). Cerebrospinal fluid chitinase 3-like 1 levels are associated with conversion to multiple sclerosis.. Brain : a journal of neurology.

[51] Yin Guo Nan, Lee Ho Won, Cho Je-Yoel, Suk Kyoungho (2009). Neuronal pentraxin receptor in cerebrospinal fluid as a potential biomarker for neurodegenerative diseases.. Brain research.

[52] Hashimoto Y, Nawa M, Kurita M, Tokizawa M, Iwamatsu A, Matsuoka M (2013). Secreted calmodulin-like skin protein inhibits neuronal death in cell-based Alzheimer's disease models via the heterotrimeric Humanin receptor.. Cell death & disease.

[53] Petit-Turcotte C, Stohl S M, Beffert U, Cohn J S, Aumont N, Tremblay M, Dea D, Yang L, Poirier J, Shachter N S (2001). Apolipoprotein C-I expression in the brain in Alzheimer's disease.. Neurobiology of disease.

[54] Plum Leona, Schubert Markus, Brüning Jens C (2005). The role of insulin receptor signaling in the brain.. Trends in endocrinology and metabolism: TEM.

[55] O'Day Danton H, Myre Michael A (2004). Calmodulin-binding domains in Alzheimer's disease proteins: extending the calcium hypothesis.. Biochemical and biophysical research communications.

